# No differences in the long-term prognosis of iris and choroidal melanomas when adjusting for tumor thickness and diameter

**DOI:** 10.1186/s12885-021-09002-0

**Published:** 2021-11-24

**Authors:** Shiva Sabazade, Christina Herrspiegel, Viktor Gill, Gustav Stålhammar

**Affiliations:** 1 St. Erik Eye Hospital, Eugeniavägen 12, 17164 Stockholm, Sweden; 2 Department of Clinical Neuroscience, Division of Eye and Vision, St. Erik Eye Hospital, Karolinska Institutet, Stockholm, Sweden; 3 Department of Pathology, Västmanland Hospital Västerås, Västerås, Sweden

**Keywords:** Melanoma, Iris melanoma, Uveal melanoma, Choroidal melanoma, Prognosis, Survival, Mortality, Tumor size

## Abstract

**Objective:**

To assess the long-term prognosis for patients with iris melanomas and compare it with the prognosis for small choroidal melanomas.

**Design:**

Retrospective observational case series.

**Methods:**

All patients treated for iris melanomas at a single referral institution between January 1st 1986 and January 1st 2016 were included. Patients treated for small choroidal melanomas during the same period were included for comparison. The cumulative incidence of melanoma-related mortality was calculated. Patient and tumor characteristics and size-adjusted hazard ratio (HR) for melanoma-related mortality were compared between iris and small choroidal melanomas.

**Results:**

Forty-five iris melanomas and 268 small choroidal melanomas were included. Twenty-four iris melanomas (53%) had been treated with local resection, 12 (27%) with Ruthenium-106 brachytherapy, 7 (16%) with enucleation and 2 (4%) with proton beam irradiation. Twenty-one (68%), 7 (16%) and 2 (4%) of the iris melanomas were of the spindle, mixed and epithelioid cell types, respectively. Twenty-three patients had deceased before the end of follow-up. Median follow-up for the 22 survivors was 13.3 years (SD 9.4). Patients with iris melanomas were more often asymptomatic at presentation and had a trend towards significantly lower age (59 versus 63 years, Student’s T-tests *p* = 0.057). Further, iris melanomas had significantly smaller basal diameter (5.8 versus 8.0 mm, *p* < 0.0001) and tumor volume (79 mm^3^ versus 93 mm mm^3^, *p* < 0.0001) but greater thickness (3.0 versus 2.5 mm, *p* < 0.0001). The cumulative incidence of iris melanoma-related mortality was 5% at 5 years after diagnosis, and 8% at 10, 15 and 20 years. The incidence was not significantly different to small choroidal melanomas (Wilcoxon *p* = 0.46). In multivariate Cox regression with tumor diameter and thickness as covariates, patients with choroidal melanomas did not have increased HR for melanoma-related mortality (HR 2.2, 95% CI 0.5–9.6, *p* = 0.29). Similarly, there were no significant survival differences in matched subgroups (Wilcoxon *p* = 0.82).

**Conclusions:**

There are no survival differences between iris and choroidal melanomas when adjusting for tumor size. The reason for the relatively favorable prognosis of iris melanomas compared to melanomas of the choroid and ciliary body is likely that they are diagnosed at a smaller size.

## Introduction

Uveal melanomas constitute 5% of all melanomas and are the most common primary intraocular malignant tumors in adults [1]. Men and women have similar incidence and survival [2]. Seeding of micrometastases occurs early, well before diagnosis of the average tumor, and treatment of the eye therefore fails to have major impact on survival [3, 4]. One third to one half of all patients develop macrometastatic disease, after which the median survival is about 1 year [5–7]. The response rate to BRAF-, CTLA-4 and PD-1 inhibition is low but other immune pathways may be targetable [8–10].

The uveal tract is a pigmented highly vascularized layer lining the inside of the sclera. In turn, the uvea is divided into three anatomical structures: The iris, the ciliary body and the choroid [11]. Uveal melanoma most commonly arises in the largest of these structures: The choroid (90% of cases), followed by the ciliary body (6%) and iris (4%) [12]. Iris melanomas are often described as having a relatively favorable prognosis and genomic features associated with ultraviolet radiation damage, whereas melanomas of the choroid and ciliary body have a higher tendency for metastatic spread and are not linked with oncogenic events that are associated with sunlight exposure or any other strong environmental or lifestyle factor [13–16].

Iris melanomas are in most cases an incidental finding due to iris color changes or pupil distortion. They may cause anterior chamber bleeding or secondary glaucoma due to compression of the anterior chamber angle, tumor invasion or obstruction of the aqueous outflow by accumulation of pigment-laden macrophages in the trabecular meshwork [17]. Iris melanomas are commonly treated with proton beam irradiation, plaque brachytherapy, local surgical resection or enucleation [15, 18]. The latter is typically reserved for eyes with large tumors that cannot be controlled with the eye-preserving alternatives. Treatment may be deferred in favor of watchful waiting and periodic photographic documentation for very small lesions [18, 19].

Previous publications indicate that the 5 and 10-year Kaplan-Meier estimates of probability for systemic metastasis for patients with iris melanomas are 4–5 and 7–9%, respectively [12, 20]. When comparing iris and choroidal melanomas in similar size increments, a slightly higher incidence of metastases has been observed for the latter. For example, the 10-year metastatic rate for iris and choroidal melanomas with a thickness of 0–3 mm has been reported to be 7 and 12%, respectively [12]. However, tumor diameter has not been taken into account, which has a greater influence on tumor volume than thickness if a semi-ellipsoid shape is assumed [21]. For example, a tumor with a thickness of 3 mm more than triples its volume if it has a diameter of 7 mm instead of 4 mm (25 versus 77 mm^3^). Further, tumor diameter has repeatedly been shown to be one of the strongest predictors of uveal melanoma-related mortality [22–24].

Therefore, we will examine the long-term prognosis of patients with iris melanoma, adjusted for both tumor thickness and diameter. The result will be compared with clinical characteristics, presenting symptoms and prognosis of patients with small choroidal melanomas, with the aim to examine if iris melanoma is truly a less aggressive tumor or if the better survival is just a matter of size.

## Materials and methods

### Patient selection

This study was approved by the Swedish Ethical Review Authority (reference 2020-02835) and adhered to the tenets of the Declaration of Helsinki. All patients who had been treated for iris melanomas at St. Erik Eye Hospital between January 1st, 1986, and January 1st, 2016, and had complete clinicopathological data available in our cancer registry were considered for the study (*n* = 45). All patients who had been treated for small choroidal melanomas (apical thickness between 1 and 3 mm and at least 5 mm but no more than 15 mm in longest basal diameter) between the same dates were included for comparison (*n* = 268).

Data on patient sex, age at diagnosis, tumor eye laterality, tumor thickness and diameter, treatment modality and survival including cause of death was retrieved from digitalized clinical records and from our digital treatment directory. At their first visit, patients were examined with slit lamp biomicroscopy and/or indirect ophthalmoscopy. A and B-scan ultrasonography and ultrasound biomicroscopy was used to measure the tumor thickness and internal reflectivity of choroidal and iris melanomas, respectively. Treatment was typically performed within 3 weeks after diagnosis.

After treatment, follow-up was scheduled at 1, 3 (patients treated with plaque brachytherapy only), 6, and 12 months and then annually for the remainder of a patient’s life. Semi-annual screening for liver metastases by ultrasonography or computed tomography was performed for 5 years after choroidal melanoma diagnosis. Thereafter, radiological examinations were only performed if prompted by patients’ symptoms.

#### Statistical analysis

Differences with a *p* value of less than 0.05 were considered statistically significant, all *p* values being two-sided. For tests of continuous variables that did not deviate significantly from normal distribution (Shapiro–Wilk test *p* > 0.05) Student’s T-tests were used. For non-parametrical data, Mann–Whitney *U* tests were used. In comparisons of categorical variables, we used two-by-two contingency tables and Pearson chi-square (χ^2^) tests (if all fields had a sample of > 5) or Fisher’s exact tests (if any field had a sample of < 5). The volume of tumors was estimated assuming a semi ellipsoid shape [4]:

$$\mathrm{Volume}\ \mathrm{of}\ \mathrm{tumor}=\frac{\uppi}{6}\times t\times {lbd}^2$$

where *t* is the tumor thickness and *lbd* is the largest basal diameter. For comparisons of survival, the cumulative incidence of melanoma-related mortality and multivariate Cox regression hazard ratios (HR) were calculated, with tumor thickness and diameter as covariates to adjust for the contribution of tumor size to outcome. We also compared matched groups of iris and choroidal melanomas. Outliers were gradually excluded to reach two groups of similar size (maximum sample difference set to 10% of the smaller group) without significant differences in patient age at diagnosis, tumor diameter and tumor thickness. To test whether our data met the proportional hazard assumption, we built a Cox regression model to calculate the HR for uveal melanoma-related mortality with a time-dependent versus a time-independent variable (tumor thickness in mm) as covariates. All statistical analyses were performed using IBM SPSS statistics version 27 (Armonk, NY).

## Results

### Descriptive statistics

Of the 45 included patients with iris melanoma, 24 were female and 21 were male. Their mean age at diagnosis was 59 years (SD 16). The mean tumor largest basal diameter was 5.8 mm (SD 3.1), and thickness 3.0 mm (SD 1.1). Twenty-four patients (53%) had been treated with local resection, 12 (27%) with Ruthenium-106 brachytherapy, 7 (16%) with enucleation and 2 (4%) with proton beam irradiation. Histopathological confirmation of the diagnosis was available for the 31 patients (69%) that had undergone local resection or enucleation. Among the 31 tumors that had been enucleated or resected, 21 (68%) were of the spindle-cell type, 7 (16%) of the mixed cell type and 2 (4%) of the epithelioid cell type. Twenty-three patients had deceased before the end of follow-up. Of these 23 dead patients, 3 had deceased from metastatic iris melanoma and 20 from other causes. Median follow-up for the 22 survivors was 13.3 years (SD 9.4, Table 1).

**Table 1:** Demographics and clinical features of included iris melanomas

***n*** =	45
**Age at diagnosis, mean (SD)**	59 (16)
**Sex, n (%)**	
Female	24 (53)
Male	21 (47)
**Mean tumor thickness, mm (SD)**	3.0 (1.1)
**Mean tumor diameter, mm (SD)**	5.8 (3.1)
**Ciliary body involvement, n (%)**	2 (4)
**Treatment modality, n (%)**	
Surgical resection	24 (53)
Plaque brachytherapy	12 (27)
Enucleation	7 (16)
Proton beam	2 (4)
**Cell type, n (%)**	
Spindle	21 (68)
Mixed	7 (23)
Epithelioid	3 (10)
**Follow-up years, median**^**a**^**(SD)**	13.3 (9.4)

*SD* Standard deviation

^a^For survivors

In comparison with the 268 patients with small choroidal melanomas, patients with iris melanomas had a trend towards significantly lower age at diagnosis (59 versus 63 years, Student’s T-tests *p* = 0.057). Further, iris melanomas had significantly smaller basal diameter (5.8 versus 8.0 mm, *p* < 0.0001) but greater thickness (3.0 versus 2.5 mm, *p* < 0.0001). No iris or choroidal melanoma had extraocular extension. The data met the proportional hazards assumption (*p* = 0.22).

### Tumor volume

The mean volume of iris melanomas was 79 mm^3^ (SD 118), which was significantly smaller than the small choroidal melanomas (93 mm^3^, SD 66, Mann-Whitney U *p* < 0.0001).

### Symptoms

Patients with iris melanoma were more often asymptomatic than patients with small choroidal melanomas. The latter were more likely to report blurred vision or decreased visual acuity on presentation (100 of 268, versus 8 of 45, χ^2^*p* = 0.05), a shadow in the visual field (38 of 268 versus 0 of 45, Fisher’s exact *p* = 0.007) and flashes and/or floaters (32 of 268 versus 0 of 45, Fisher’s exact *p* = 0.02). There were no significant differences in the distribution of pain (2 of 268 versus 0 of 45, Fisher’s exact *p* = 1.0) or metamorphopsias (0 of 45 versus 3 of 268, Fisher’s exact *p* = 0.23).

### Prognosis

**Fig. 1:**
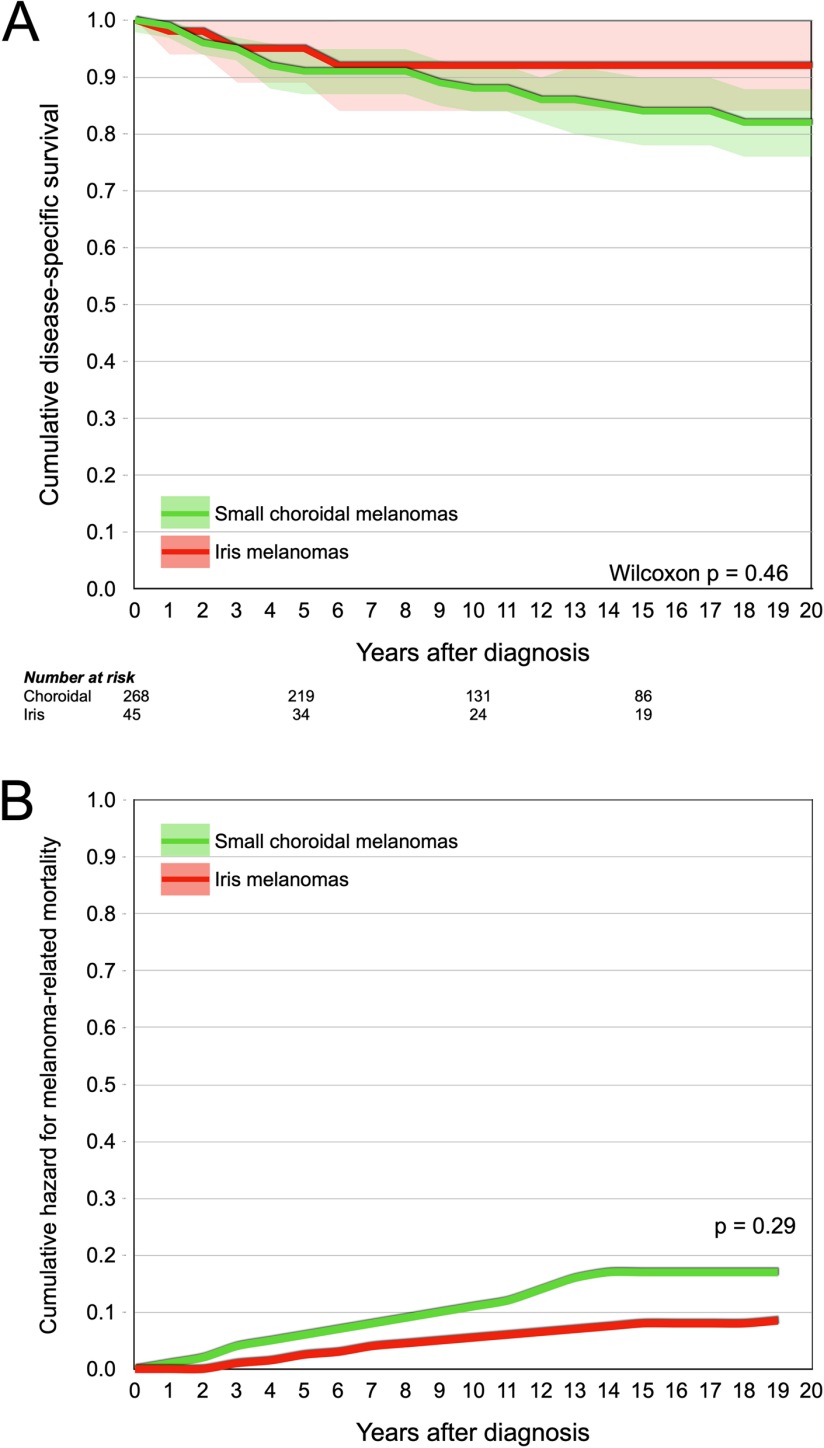
**A** Survival curve. Cumulative disease-specific survival for patients with small choroidal (green) and iris (red) melanomas. The difference was not significant (Wilcoxon *p* = 0.46). **B** Multivariate Cox regression. Cumulative hazard ratio for melanoma-related mortality for choroidal (green) versus iris (red) melanomas, adjusted for tumor thickness and diameter. The hazard ratio was not significantly greater for choroidal melanomas (HR 2.2, 95% CI 0.5–9.6, *p* = 0.29)

The cumulative incidence of iris melanoma-related mortality was 5% at 5 years after diagnosis, and 8% at 10, 15 and 20 years. The cumulative incidence of choroidal melanoma-related mortality was 9% at 5 years after diagnosis, 12% at 10 years, 16% at 15 years and 18% at 20 years. The difference was not significant (Wilcoxon *p* = 0.46, Fig. 1a).

In multivariate Cox regression with tumor diameter and thickness as covariates, patients with choroidal melanomas did not have increased HR for melanoma-related mortality (HR 2.2, 95% CI 0.5–9.6, *p* = 0.29, Fig. 1b). Both tumor diameter (HR 1.1 per increased mm, 95% CI 1.0–1.3, *p* = 0.021) and thickness (HR 1.7 per increased mm, 95% CI 1.0–2.8, *p* = 0.042) were independent predictors.

### Matched cohorts

**Fig. 2:**
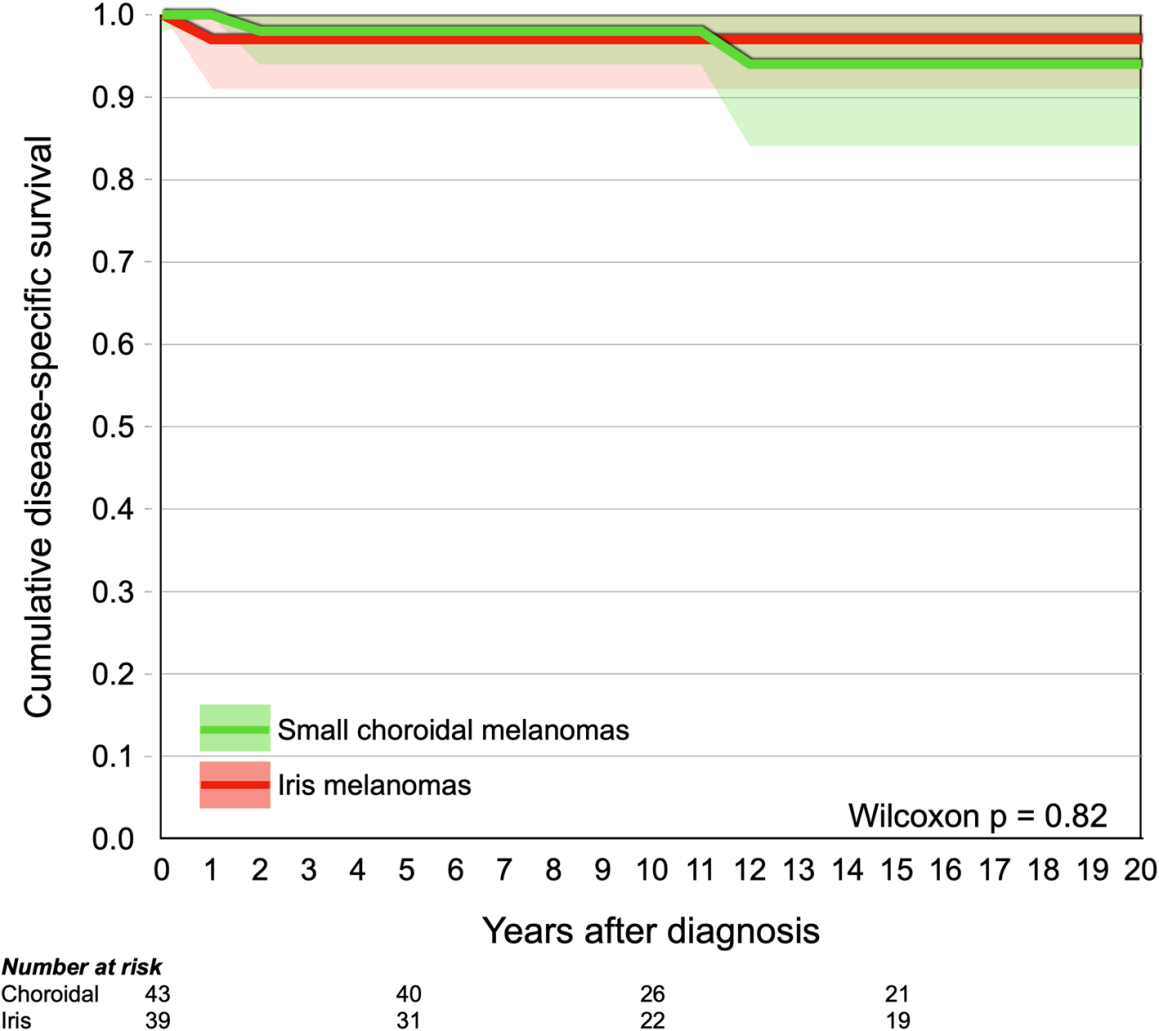
Survival curve, cumulative disease-specific survival for patients with small choroidal (green) and iris (red) melanomas, matched for age at diagnosis, tumor thickness and diameter. The difference was not significant (Wilcoxon *p* = 0.82)

Thirty-nine iris melanomas were matched with 43 choroidal melanomas based on patient age at diagnosis, tumor diameter and tumor thickness. Two of the iris melanomas and one of the choroidal melanomas involved the ciliary body (Fisher’s exact *p* = 0.60). No comparison of tumor cell type was possible since none of the choroidal melanomas had been enucleated or biopsied. The included patients with choroidal melanoma more often presented with a shadow in the visual field (Table 2). Again, there was no significant difference in cumulative melanoma-related mortality (Wilcoxon *p* = 0.82, Fig. 2).

**Table 2:** Characteristics of patients and tumors in matched subgroups. SD, standard deviation. IQR, interquartile range

	Iris melanomas (***n*** = 39)	Choroidal melanomas (***n*** = 43)	***p***
**Patient age at diagnosis, mean (SD)**	58 (17)	63 (13)	0.20
**Tumor diameter, mean (SD)**	5.3 (2.9)	5.3 (1.0)	0.93
**Tumor thickness, mean (SD)**	2.8 (0.8)	2.6 (0.3)	0.34
**Ciliary body involvement, n (%)**	2 (5)	1 (2)	0.60
**Presenting symptoms, n (%)**			
Blurred vision or decreased visual acuity	6 (15)	13 (30)	0.21
Shadow in visual field	0 (0)	10 (23)	0.004
Flashes and/or floaters	0 (0)	3 (7)	0.25
Metamorphopsia	0 (0)	0 (0)	1.0
Ocular pain	0 (0)	0 (0)	1.0
**Median follow up for survivors, years (IQR)**	14.0 (14.5)	15.2 (13.9)	0.54

## Discussion

In this study, we have shown that there are no significant differences in melanoma-related mortality between patients with melanoma of the iris and choroid when adjusting for tumor size and diameter – regardless of comparisons with regression analysis or in matched cohorts. This indicates that iris melanomas do not have intrinsic properties that make them a more benign type of uveal melanoma. Rather, they are associated with a relatively good prognosis only because they are diagnosed at an earlier stage than the more hidden melanomas of the choroid or ciliary body. Our observed cumulative mortality rates of 5 and 8% at 5 and 10 years, respectively virtually matched previous published Kaplan-Meier estimates of probability for metastatic disease from iris melanomas with similar size [12, 20]. However, we used melanoma-related mortality as an endpoint, which occurs about 1 year after metastasis [7, 25]. The effect of this difference may have been offset by us using cumulative incidence instead of Kaplan-Meier as the latter is known to overestimate the incidence in presence of competing risks (i.e. death from other causes before onset of metastases).

In turn, this highlights two important perspectives. First, the previously published observation that iris melanomas, in contrast to choroidal and ciliary body melanomas, display genomic features associated with ultraviolet radiation damage apparently does not lead to any obvious differences in metastatic risk [13]. Second, the low 20-year mortality rates for patients with small iris and choroidal melanomas may indicate that primary tumor treatment has some effect on survival after all. This is a debated topic [3]. In short, large tumors have a high risk of metastasis and small tumors have a low risk. All large tumors were once small, but all small tumors may not necessarily grow large and acquire monosomy 3, *BAP1* mutations, vasculogenic mimicry or other high-risk features even if left untreated [3]. Some tumors seem to have these features from the onset, and there is marked variability in the growth rate of uveal melanomas as well as intratumor heterogeneity of risk factors [4, 26–30]. Nonetheless, there is no doubt that the likelihood of high-risk features increases with increasing tumor size [4, 31–33]. Consequently, if all tumors were small at treatment, the ones that would have developed high-risk features later would be included, thus reducing metastatic rates. This may be a utopia for choroidal and ciliary body melanomas that are often asymptomatic until they have become medium-sized or even large but is perfectly illustrated by iris melanomas: They are treated when they are small, and metastatic rates are low. This conclusion may be supported by previous indications of increased mortality among patients who chose to defer or receive no treatment, whereas it is contradicted by other studies that have found no increased mortality when treatment is delayed [34, 35].

Choroidal melanomas typically present with blurred vision, a shadow in the visual field, photopsias or floaters [36]. The fact that patients with iris melanomas in the present study generally had fewer symptoms is likely relatable to their anatomical location, with less disturbance of the retina and less potential for exudative retinal detachment.

There are several limitations to this study. The number of patients with iris melanomas was rather small. They were included from one institution only and the data was retrospective and non-randomized, which limits our control over confounding factors. We had no data from sequencing studies or immunohistochemical, histological or fluorescence in situ hybridization examinations, which would have helped us investigate similarities and differences between iris and choroidal melanomas further. For example, we cannot fully exclude that there is a difference in the proportion of tumors with *BAP1* mutations or vasculogenic mimicry, which in turn could have led significant differences in survival had the cohort been larger.

In conclusion, this comparison of patients with iris and small choroidal melanomas revealed no differences in long-term survival. The reason for the relatively favorable prognosis in iris melanoma is likely that they are diagnosed at a smaller size. In other words: Prognostically, iris melanoma is just another uveal melanoma. Future studies should include more patients with larger iris melanomas, and ideally, observations of the natural course of untreated tumors.

## Data Availability

The raw datasets generated and/or analyzed during the current study are not publicly available as the authors have no legal or ethical right to disclose protected health information, but anonymized data are available from the corresponding author on reasonable request.

## Abbreviations

CI: Confidence interval
HR: Hazard ratio
IQR: Interquartile range
*lbd*: Largest basal diameter
mm: Millimeter
SD: Standard deviation
*t*: Tumor thickness
