# Correction to: No differences in the long-term prognosis of iris and choroidal melanomas when adjusting for tumor thickness and diameter

**DOI:** 10.1186/s12885-021-09167-8

**Published:** 2022-01-04

**Authors:** Shiva Sabazade, Christina Herrspiegel, Viktor Gill, Gustav Stålhammar

**Affiliations:** 1 St. Erik Eye Hospital, Eugeniavägen 12, 17164 Stockholm, Sweden; 2 Department of Clinical Neuroscience, Division of Eye and Vision, St. Erik Eye Hospital, Karolinska Institutet, Stockholm, Sweden; 3 Department of Pathology, Västmanland Hospital Västerås, Västerås, Sweden

**Correction to: BMC Cancer 21, 1270 (2021)**

**https://doi.org/10.1186/s12885-021-09002-0**

Following publication of the original article [1], the authors noticed an error in their article.

The third sentence of the Results section of the abstract: “Twenty-one (68%), 7 (16%) and 2 (4%) of the iris melanomas were of the spindle, mixed and epithelioid cell types, respectively.” Should be replaced with: “Twenty-one (68%), 7 (23%) and 3 (10%) of the surgically treated iris melanomas were of the spindle, mixed and epithelioid cell types, respectively.”

Similarly, the same sentence should replace the sixth sentence in the Descriptive statistics of the main text.

The numbers are correctly stated in Table 1.
